# Supplementary material for: Real-world patterns in remote longitudinal study participation: A study of the Swiss Multiple Sclerosis Registry
Source: PLOS Digit Health. 2024 Nov 6;3(11):e0000645. doi: 10.1371/journal.pdig.0000645 (PMC11540223; doi:10.1371/journal.pdig.0000645)
Supplement: S1 Fig — (DOCX) [file pdig.0000645.s001.docx]

## **S1 Fig.** Flowchart of included participants

*Minimal requirements for study inclusion were complete data on age, living situation, education, citizenship, partnership

L2A2 and L3A3 online baseline participants (**n=2314**, **n=1817**)

Filter for participants who filled out both L2A2 and L3A3 questionnaires

Online baseline participants (**n=1817**)

Filter for participants with complete socio-

demographic data*

Online baseline participants (**n=1757**)

Filter for complete

cases

Complete cases (**n=1602**)
