# Supplementary figures and images for: Real-world patterns in remote longitudinal study participation: A study of the Swiss Multiple Sclerosis Registry
Source: PLOS Digit Health. 2024 Nov 6;3(11):e0000645. doi: 10.1371/journal.pdig.0000645 (PMC11540223; doi:10.1371/journal.pdig.0000645)

## **S2 Fig**: Elbow method


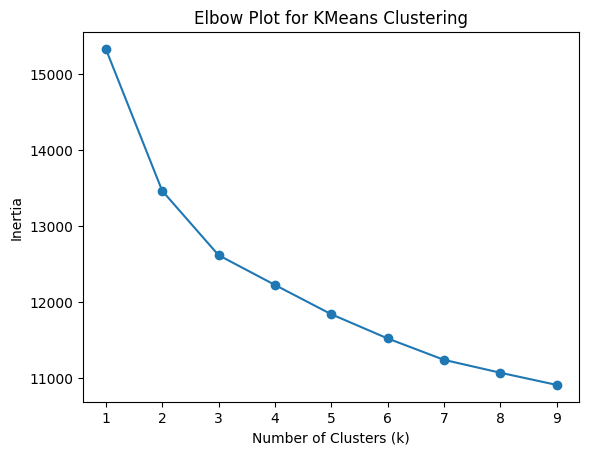

Supplement: S2 Fig — (DOCX) [file pdig.0000645.s002.docx]

## **S3 Fig**: Sankey plots of clustering results

***
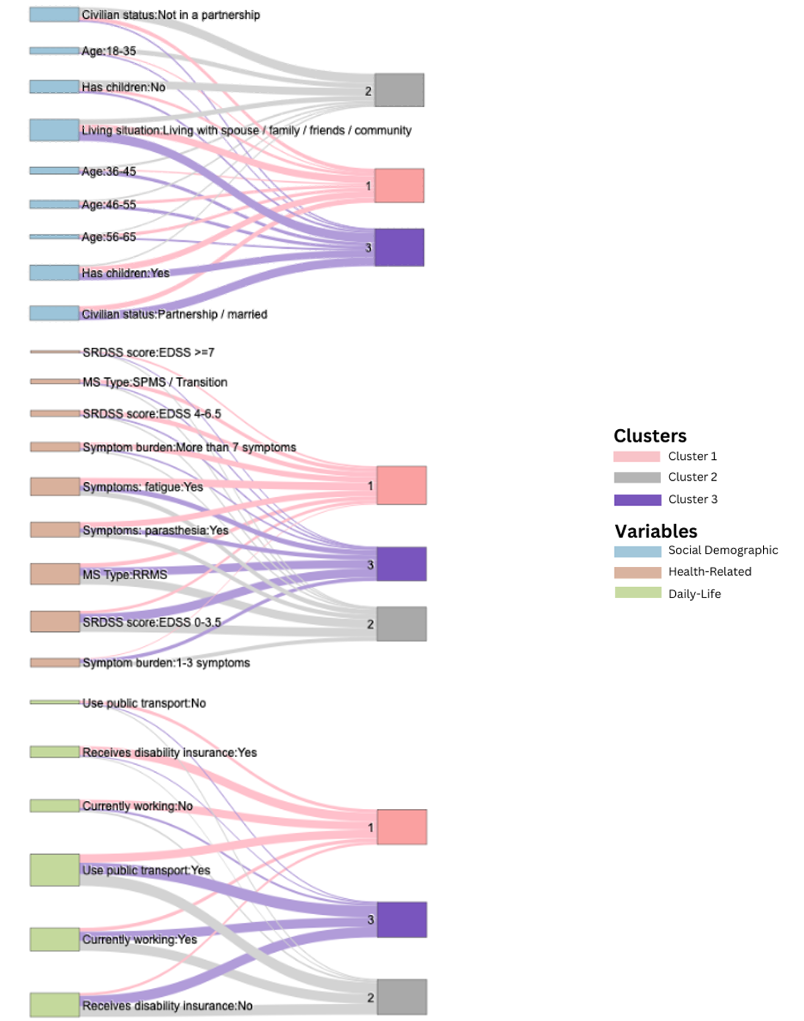
***

Supplement: S3 Fig — (DOCX) [file pdig.0000645.s003.docx]
