# Supplementary material for: Real-world patterns in remote longitudinal study participation: A study of the Swiss Multiple Sclerosis Registry
Source: PLOS Digit Health. 2024 Nov 6;3(11):e0000645. doi: 10.1371/journal.pdig.0000645 (PMC11540223; doi:10.1371/journal.pdig.0000645)
Supplement: S4 Fig — (DOCX) [file pdig.0000645.s004.docx]

## **S4 Fig:** Flesch-Kinkaid score and impact on non-completion, with filtered out survey completers who answered less than the median of survey questions
