# Supplementary material for: Real-world patterns in remote longitudinal study participation: A study of the Swiss Multiple Sclerosis Registry
Source: PLOS Digit Health. 2024 Nov 6;3(11):e0000645. doi: 10.1371/journal.pdig.0000645 (PMC11540223; doi:10.1371/journal.pdig.0000645)
Supplement: S1 Table — (DOCX) [file pdig.0000645.s005.docx]

## **S1 Table**: Yearly retention and starting year-based retention outcomes for participants who deregistered from the SMSR or died during the study

|  | **Yearly retention** | **Starting year-based retention** |
| --- | --- | --- |
| High | 14/41 (34%) | 13/41 (32% |
| Low | 27/41 (66%) | 28/41 (68%) |
