# Supplementary material for: Real-world patterns in remote longitudinal study participation: A study of the Swiss Multiple Sclerosis Registry
Source: PLOS Digit Health. 2024 Nov 6;3(11):e0000645. doi: 10.1371/journal.pdig.0000645 (PMC11540223; doi:10.1371/journal.pdig.0000645)
Supplement: S2 Table — (DOCX) [file pdig.0000645.s006.docx]

## **S2 Table**: Sensitivity analysis with alternate outcome variable, participant characteristics, yearly retention until the end of the study period

|  | **Low retention**  **(N=1332)** | **High retention**  **(N=425)** | **Overall (N=1757)** |
| --- | --- | --- | --- |
| **Age** |  |  |  |
| 18-35 | 355 (26.7%) | 73 (17.2%) | 428 (24.4%) |
| 36-45 | 348 (26.1%) | 116 (27.3%) | 464 (26.4%) |
| 46-55 | 371 (27.9%) | 136 (32.0%) | 507 (28.9%) |
| 56-65 | 182 (13.7%) | 78 (18.4%) | 260 (14.8%) |
| 66 and older | 76 (5.7%) | 22 (5.2%) | 98 (5.6%) |
| **Sex** |  |  |  |
| Male | 391 (29.4%) | 112 (26.4%) | 503 (28.6%) |
| Female | 941 (70.6%) | 313 (73.6%) | 1254 (71.4%) |
| **Language region** |  |  |  |
| German / Romansch | 1005 (75.5%) | 352 (82.8%) | 1357 (77.2%) |
| French | 232 (17.4%) | 59 (13.9%) | 291 (16.6%) |
| Italian | 48 (3.6%) | 10 (2.4%) | 58 (3.3%) |
| Missing | 47 (3.5%) | 4 (0.9%) | 51 (2.9%) |
| **Survey start year** |  |  |  |
| 2016 | 376 (28.2%) | 158 (37.2%) | 534 (30.4%) |
| 2017-2019 | 723 (54.3%) | 200 (47.1%) | 923 (52.5%) |
| 2020 onwards | 233 (17.5%) | 67 (15.8%) | 300 (17.1%) |
| **Has children** |  |  |  |
| Yes | 709 (53.2%) | 243 (57.2%) | 952 (54.2%) |
| No | 623 (46.8%) | 182 (42.8%) | 805 (45.8%) |
| **Highest degree: university** |  |  |  |
| Yes | 420 (31.5%) | 136 (32.0%) | 556 (31.6%) |
| No | 912 (68.5%) | 289 (68.0%) | 1201 (68.4%) |
| **Civilian status** |  |  |  |
| Partnership / married | 665 (49.9%) | 241 (56.7%) | 906 (51.6%) |
| Not in a partnership | 667 (50.1%) | 184 (43.3%) | 851 (48.4%) |
| **Living situation** |  |  |  |
| Living with spouse / family / friends / community | 1016 (76.3%) | 339 (79.8%) | 1355 (77.1%) |
| Living alone / Single-parenting | 316 (23.7%) | 86 (20.2%) | 402 (22.9%) |
| **Swiss citizenship** |  |  |  |
| Yes | 1159 (87.0%) | 389 (91.5%) | 1548 (88.1%) |
| No | 173 (13.0%) | 36 (8.5%) | 209 (11.9%) |
| **Years since MS diagnosis** |  |  |  |
| Mean (SD) | 8.88 (8.94) | 9.04 (8.55) | 8.92 (8.85) |
| Median [Min, Max] | 6.00 [0, 49.0] | 7.00 [0, 39.0] | 6.00 [0, 49.0] |
| Missing | 34 (2.6%) | 11 (2.6%) | 45 (2.6%) |
| **MS Type** |  |  |  |
| RRMS | 962 (72.2%) | 295 (69.4%) | 1257 (71.5%) |
| CIS | 36 (2.7%) | 10 (2.4%) | 46 (2.6%) |
| PPMS | 124 (9.3%) | 37 (8.7%) | 161 (9.2%) |
| SPMS / Transition | 186 (14.0%) | 76 (17.9%) | 262 (14.9%) |
| Missing | 24 (1.8%) | 7 (1.6%) | 31 (1.8%) |
| RRMS | 962 (72.2%) | 295 (69.4%) | 1257 (71.5%) |
| **MS in relatives** |  |  |  |
| Yes | 257 (19.3%) | 88 (20.7%) | 345 (19.6%) |
| No | 1075 (80.7%) | 337 (79.3%) | 1412 (80.4%) |
| **Symptoms: fatigue** |  |  |  |
| Yes | 801 (60.1%) | 244 (57.4%) | 1045 (59.5%) |
| No | 531 (39.9%) | 181 (42.6%) | 712 (40.5%) |
| **Symptoms: paresthesia** |  |  |  |
| Yes | 650 (48.8%) | 229 (53.9%) | 879 (50.0%) |
| No | 682 (51.2%) | 196 (46.1%) | 878 (50.0%) |
| **Symptoms: depression** |  |  |  |
| Yes | 182 (13.7%) | 42 (9.9%) | 224 (12.7%) |
| No | 1150 (86.3%) | 383 (90.1%) | 1533 (87.3%) |
| **Symptom burden** |  |  |  |
| 1-3 symptoms | 412 (30.9%) | 134 (31.5%) | 546 (31.1%) |
| 4-6 symptoms | 329 (24.7%) | 121 (28.5%) | 450 (25.6%) |
| More than 7 symptoms | 391 (29.4%) | 114 (26.8%) | 505 (28.7%) |
| No symptoms | 200 (15.0%) | 56 (13.2%) | 256 (14.6%) |
| **SRDSS score** |  |  |  |
| SRDSS 0-3.5 | 984 (73.9%) | 311 (73.2%) | 1295 (73.7%) |
| SRDSS 4-6.5 | 229 (17.2%) | 86 (20.2%) | 315 (17.9%) |
| SRDSS >=7 | 92 (6.9%) | 17 (4.0%) | 109 (6.2%) |
| Missing | 27 (2.0%) | 11 (2.6%) | 38 (2.2%) |
| **Receives disability insurance** |  |  |  |
| Yes | 387 (29.1%) | 123 (28.9%) | 510 (29.0%) |
| No | 945 (70.9%) | 302 (71.1%) | 1247 (71.0%) |
| **Currently drives** |  |  |  |
| Yes | 1034 (77.6%) | 342 (80.5%) | 1376 (78.3%) |
| No | 298 (22.4%) | 83 (19.5%) | 381 (21.7%) |
| **Use public transport** |  |  |  |
| Yes | 1188 (89.2%) | 389 (91.5%) | 1577 (89.8%) |
| No | 144 (10.8%) | 36 (8.5%) | 180 (10.2%) |
| **Currently working** |  |  |  |
| Yes | 887 (66.6%) | 286 (67.3%) | 1173 (66.8%) |
| No | 445 (33.4%) | 139 (32.7%) | 584 (33.2%) |
| **Someone helped with survey** |  |  |  |
| Yes | 73 (5.5%) | 16 (3.8%) | 89 (5.1%) |
| No | 1259 (94.5%) | 409 (96.2%) | 1668 (94.9%) |
