# Supplementary material for: Real-world patterns in remote longitudinal study participation: A study of the Swiss Multiple Sclerosis Registry
Source: PLOS Digit Health. 2024 Nov 6;3(11):e0000645. doi: 10.1371/journal.pdig.0000645 (PMC11540223; doi:10.1371/journal.pdig.0000645)
Supplement: S3 Table — (DOCX) [file pdig.0000645.s007.docx]

## **S3 Table**: Univariate and multivariable logistic regression, yearly retention

| **Variable** | **Univariate** | | **Multivariable - Global** | | **Multivariable - Imputed** | |
| --- | --- | --- | --- | --- | --- | --- |
|  | **OR***^1^* | **95% CI***^1^* | **OR***^1^* | **95% CI***^1^* | **OR***^1^* | **95% CI***^1^* |
| **Age** |  |  |  |  |  |  |
| 18-35 | — | — | — | — | — | — |
| 36-45 | **1.40** | **1.04, 1.87** | **1.60** | **1.16, 2.21** | **1.45** | **1.07, 1.97** |
| 46-55 | **1.53** | **1.15, 2.03** | **1.58** | **1.13, 2.20** | **1.48** | **1.08, 2.04** |
| 56-65 | **1.45** | **1.03, 2.03** | 1.42 | 0.92, 2.19 | 1.41 | 0.94, 2.12 |
| 66 and older | 1.30 | 0.78, 2.13 | 0.98 | 0.50, 1.89 | 1.00 | 0.55, 1.82 |
| **Sex** |  |  |  |  |  |  |
| Male | — | — | — | — | — | — |
| Female | 1.02 | 0.82, 1.28 | 1.11 | 0.87, 1.42 | 1.11 | 0.88, 1.40 |
| **Language region** |  |  |  |  |  |  |
| German / Romansch | — | — | — | — | — | — |
| French | **0.74** | **0.56, 0.98** | 0.76 | 0.56, 1.03 | 0.78 | 0.58, 1.04 |
| Italian | 0.69 | 0.38, 1.23 | 0.76 | 0.40, 1.38 | 0.89 | 0.50, 1.56 |
| **Survey start year** |  |  |  |  |  |  |
| 2016 | — | — | — | — | — | — |
| 2017-2019 | **0.74** | **0.59, 0.92** | **0.77** | **0.61, 0.97** | **0.78** | **0.62, 0.97** |
| 2020 onwards | **0.42** | **0.30, 0.59** | **0.40** | **0.28, 0.57** | **0.41** | **0.30, 0.57** |
| **Has children** |  |  |  |  |  |  |
| No | — | — | — | — | — | — |
| Yes | 1.10 | 0.90, 1.35 | 0.98 | 0.77, 1.27 | 1.00 | 0.79, 1.27 |
| **Highest degree: (applied) university** |  |  |  |  |  |  |
| No | — | — | — | — | — | — |
| Yes | 1.05 | 0.84, 1.30 | 1.09 | 0.86, 1.37 | 1.15 | 0.92, 1.44 |
| **Civil status** |  |  |  |  |  |  |
| Not in a partnership | — | — | — | — | — | — |
| Partnership / married | 1.16 | 0.94, 1.42 | 0.96 | 0.72, 1.27 | 1.01 | 0.77, 1.32 |
| **Living situation** |  |  |  |  |  |  |
| Living alone / Single-parenting | — | — | — | — | — | — |
| Living with spouse / family / friends / community | 1.18 | 0.92, 1.51 | 1.19 | 0.87, 1.63 | 1.11 | 0.83, 1.50 |
| **Swiss citizenship** |  |  |  |  |  |  |
| No | — | — | — | — | — | — |
| Yes | **1.73** | **1.22, 2.50** | **1.60** | **1.11, 2.34** | **1.66** | **1.19, 2.35** |
| **Years since MS diagnosis** | 1.00 | 0.99, 1.01 | 1.00 | 0.98, 1.01 | 0.99 | 0.87, 1.13 |
| **MS type** |  |  |  |  |  |  |
| RRMS | — | — | — | — | — | — |
| CIS | 1.42 | 0.66, 3.03 | 1.60 | 0.72, 3.49 | 0.96 | 0.50, 1.81 |
| PPMS | 0.96 | 0.66, 1.36 | 0.98 | 0.65, 1.48 | 1.14 | 0.78, 1.68 |
| SPMS / Transition | **1.40** | **1.04, 1.86** | **1.57** | **1.08, 2.28** | **1.64** | **1.16, 2.33** |
| **MS in relatives** |  |  |  |  |  |  |
| No | — | — | — | — | — | — |
| Yes | 0.99 | 0.77, 1.28 | 1.00 | 0.77, 1.29 | 0.94 | 0.73, 1.20 |
| **Symptoms: fatigue** |  |  |  |  |  |  |
| No | — | — | — | — | — | — |
| Yes | 0.83 | 0.67, 1.02 | 0.79 | 0.60, 1.05 | 0.89 | 0.68, 1.16 |
| **Symptoms: paresthesia** |  |  |  |  |  |  |
| No | — | — | — | — | — | — |
| Yes | 1.14 | 0.93, 1.39 | 1.19 | 0.92, 1.53 | 1.21 | 0.95, 1.54 |
| **Symptoms: depression** |  |  |  |  |  |  |
| No | — | — | — | — | — | — |
| Yes | **0.72** | **0.52, 0.98** | 0.81 | 0.57, 1.16 | 0.80 | 0.57, 1.11 |
| **Symptom burden** |  |  |  |  |  |  |
| No symptoms | — | — | — | — | — | — |
| 1-3 symptoms | 0.91 | 0.66, 1.26 | 0.92 | 0.62, 1.35 | 0.83 | 0.57, 1.19 |
| 4-6 symptoms | 1.18 | 0.85, 1.65 | 1.25 | 0.79, 1.99 | 1.04 | 0.68, 1.62 |
| More than 7 symptoms | 0.87 | 0.63, 1.22 | 0.96 | 0.55, 1.66 | 0.80 | 0.48, 1.35 |
| **SRDSS score** |  |  |  |  |  |  |
| EDSS 0-3.5 | — | — | — | — | — | — |
| EDSS 4-6.5 | 1.22 | 0.94, 1.58 | 1.27 | 0.91, 1.82 | 1.28 | 0.92, 1.79 |
| EDSS >=7 | 0.84 | 0.54, 1.28 | 0.90 | 0.49, 1.63 | 0.98 | 0.56, 1.70 |
| **Receives disability insurance** |  |  |  |  |  |  |
| No | — | — | — | — | — | — |
| Yes | 0.86 | 0.68, 1.07 | **0.66** | **0.49, 0.90** | **0.73** | **0.55, 0.97** |
| **Currently drives** |  |  |  |  |  |  |
| No | — | — | — | — | — | — |
| Yes | 1.13 | 0.88, 1.47 | 1.07 | 0.81, 1.43 | 1.04 | 0.80, 1.36 |
| **Uses public transport** |  |  |  |  |  |  |
| No | — | — | — | — | — | — |
| Yes | 1.20 | 0.84, 1.73 | 1.39 | 0.88, 2.20 | 1.33 | 0.88, 2.01 |
| **Currently working** |  |  |  |  |  |  |
| No | — | — | — | — | — | — |
| Yes | 0.93 | 0.75, 1.16 | 0.81 | 0.62, 1.07 | 0.87 | 0.67, 1.12 |
| **Someone helped with survey** |  |  |  |  |  |  |
| No | — | — | — | — | — | — |
| Yes | 1.08 | 0.67, 1.72 | 1.18 | 0.70, 1.98 | 1.20 | 0.74, 1.92 |
