# Supplementary material for: Real-world patterns in remote longitudinal study participation: A study of the Swiss Multiple Sclerosis Registry
Source: PLOS Digit Health. 2024 Nov 6;3(11):e0000645. doi: 10.1371/journal.pdig.0000645 (PMC11540223; doi:10.1371/journal.pdig.0000645)
Supplement: S5 Table — (DOCX) [file pdig.0000645.s009.docx]

## **S5 Table**: Clustering task, baseline participants

| **Variable** | **Cluster 1 (n=456)** | **Cluster 2 (n=538)** | **Cluster 3 (n=608)** |
| --- | --- | --- | --- |
| **Age** |  |  |  |
| 18-35 | 13 (2.9%) | 278 (51.7%) | 106 (17.4%) |
| 36-45 | 71 (15.6%) | 130 (24.2%) | 220 (36.2%) |
| 46-55 | 152 (33.3%) | 102 (24.2%) | 211 (36.2%) |
| 56-65 | 159 (34.9%) | 22 (4.1%) | 57 (9.4%) |
| 66 and older | 61 (13.4%) | 6 (1.1%) | 14 (2.3%) |
| **Sex** |  |  |  |
| Male | 175 (7.9%) | 97 (12.4%) | 182 (29.9%) |
| Female | 281 (61.6%) | 441 (82.0%) | 426 (70.1%) |
| **Language region** |  |  |  |
| German / Romansch | 358 (78.5%) | 441 (82.0%) | 471 (77.5%) |
| French | 88 (19.3%) | 74 (13.8%) | 115 (18.9%) |
| Italian | 10 (2.2%) | 23 (4.3%) | 22 (3.6%) |
| **Survey start year** |  |  |  |
| 2016 | 174 (38.2%) | 139 (25.8%) | 187 (30.8%) |
| 2017-2019 | 242 (53.1%) | 277 (51.5%) | 310 (51.0%) |
| 2020 onwards | 40 (8.8%) | 122 (22.7%) | 111 (18.3%) |
| **Has children** |  |  |  |
| No | 144 (31.6%) | 447 (83.1%) | 145 (23.9%) |
| Yes | 312 (68.4%) | 91 (16.9%) | 463 (76.2%) |
| **Highest degree: (applied) university** |  |  |  |
| No | 346 (75.9%) | 353 (65.6%) | 396 (65.1%) |
| Yes | 110 (24.1%) | 185 (34.4%) | 212 (34.9%) |
| **Civil status** |  |  |  |
| Not in a partnership | 200 (43.9%) | 538 (100.0%) | 37 (6.1%) |
| Partnership / married | 256 (56.1%) | 0 (0.0%) | 571 (93.9%) |
| **Living situation** |  |  |  |
| Living alone / Single-parenting | 108 (23.7%) | 248 (46.1%) | 2 (0.3%) |
| Living with spouse / family / friends / community | 348 (76.3%) | 290 (53.9%) | 606 (99.7%) |
| **Swiss citizenship** |  |  |  |
| No | 36 (7.9%) | 67 (12.5%) | 70 (11.5%) |
| Yes | 420 (92.1%) | 471 (87.6%) | 538 (88.5%) |
| **Years since MS diagnosis** | 17 (10) | 2.0 (6.0) | 4.0 (9.0) |
| **MS type** |  |  |  |
| RRMS | 193 (42.3%) | 476 (88.5%) | 525 (86.4%) |
| CIS | 3 (0.7%) | 20 (3.7%) | 7 (1.2%) |
| PPMS | 76 (16.7%) | 22 (4.1%) | 51 (8.4%) |
| SPMS / Transition | 184 (40.4%) | 20 (3.7%) | 25 (4.1%) |
| **MS in relatives** |  |  |  |
| No | 364 (79.8%) | 429 (79.7%) | 488 (80.3%) |
| Yes | 92 (20.2%) | 109 (20.3%) | 120 (19.7%) |
| **Symptoms: fatigue** |  |  |  |
| No | 95 (20.8%) | 260 (48.3%) | 282 (46.4%) |
| Yes | 361 (79.2%) | 278 (51.7%) | 326 (53.6%) |
| **Symptoms: paresthesia** |  |  |  |
| No | 174 (38.2%) | 288 (53.5%) | 333 (54.8%) |
| Yes | 282 (61.8%) | 250 (46.7%) | 275 (45.2%) |
| **Symptoms: depression** |  |  |  |
| No | 370 (81.1%) | 465 (86.4%) | 564 (92.8%) |
| Yes | 86 (18.9%) | 73 (13.6%) | 44 (7.2%) |
| **Symptom burden** |  |  |  |
| No symptoms | 4 (0.88%) | 112 (20.8%) | 112 (18.4%) |
| 1-3 symptoms | 47 (10.3%) | 222 (41.3%) | 231 (38.0%) |
| 4-6 symptoms | 143 (31.4%) | 114 (21.2%) | 153 (25.2%) |
| More than 7 symptoms | 262 (57.5%) | 90 (16.7%) | 112 (18.4%) |
| **SRDSS score** |  |  |  |
| SRDSS 0-3.5 | 140 (13.4%) | 508 (1.12%) | 559 (91.9%) |
| SRDSS 4-6.5 | 225 (49.3%) | 25 (4.65%) | 45 (7.4%) |
| SRDSS >=7 | 91 (20.0%) | 5 (0.93%) | 4 (0.66%) |
| **Receives disability insurance** |  |  |  |
| No | 107 (23.5%) | 486 (90.3%) | 544 (89.5%) |
| Yes | 349 (76.5%) | 52 (9.7%) | 64 (10.5%) |
| **Currently drives** |  |  |  |
| No | 159 (34.9%) | 98 (18.2%) | 68 (11.2%) |
| Yes | 297 (65.1%) | 440 (81.8%) | 540 (88.8%) |
| **Uses public transport** |  |  |  |
| No | 129 (28.3%) | 6 (1.1%) | 9 (1.5%) |
| Yes | 327 (71.7%) | 532 (98.9%) | 599 (98.5%) |
| **Currently working** |  |  |  |
| No | 314 (68.9%) | 86 (16.0%) | 119 (19.6%) |
| Yes | 142 (31.1%) | 452 (84.0%) | 489 (80.4%) |
| **Someone helped with survey** |  |  |  |
| No | 419 (91.9%) | 529 (98.3%) | 578 (95.1%) |
| Yes | 37 (8.1%) | 9 (1.7%) | 30 (4.9%) |
