# Supplementary material for: Real-world patterns in remote longitudinal study participation: A study of the Swiss Multiple Sclerosis Registry
Source: PLOS Digit Health. 2024 Nov 6;3(11):e0000645. doi: 10.1371/journal.pdig.0000645 (PMC11540223; doi:10.1371/journal.pdig.0000645)
Supplement: S6 Table — (DOCX) [file pdig.0000645.s010.docx]

## **S6 Table**: Retention outcomes based on identified clusters

| **Cluster** | **Retention outcomes** | **Percentage** |
| --- | --- | --- |
| Cluster 1 (n = 456) | Low retention | 59.2 |
|  | High retention | 40.8 |
|  | Lower response than median | 47.8 |
|  | Higher response than median | 52.2 |
| Cluster 2 (n = 538) | Low retention | 63.4 |
|  | High retention | 36.6 |
|  | Lower response than median | 44.8 |
|  | Higher response than median | 55.2 |
| Cluster 3 (n = 608) | Low retention | 61.2 |
|  | High retention | 38.8 |
|  | Lower response than median | 44.3 |
|  | Higher response than median | 56.7 |
