# Supplementary material for: Real-world patterns in remote longitudinal study participation: A study of the Swiss Multiple Sclerosis Registry
Source: PLOS Digit Health. 2024 Nov 6;3(11):e0000645. doi: 10.1371/journal.pdig.0000645 (PMC11540223; doi:10.1371/journal.pdig.0000645)
Supplement: S7 Table — (DOCX) [file pdig.0000645.s011.docx]

## **S7 Table**: Univariate and multivariable logistic regression, starting year-based retention

| **Variable** | **Univariate** | | **Multivariable - Global** | | **Multivariable - Imputed** | |
| --- | --- | --- | --- | --- | --- | --- |
|  | **OR***^1^* | **95% CI***^1^* | **OR***^1^* | **95% CI***^1^* | **OR***^1^* | **95% CI***^1^* |
| **Age** |  |  |  |  |  |  |
| 18-35 | — | — | — | — | — | — |
| 36-45 | **1.52** | **1.17, 1.98** | **2.01** | **1.48, 2.74** | **1.84** | **1.37, 2.47** |
| 46-55 | **1.50** | **1.16, 1.94** | **2.11** | **1.53, 2.92** | **1.96** | **1.44, 2.69** |
| 56-65 | **1.42** | **1.05, 1.94** | **2.17** | **1.44, 3.30** | **2.11** | **1.42, 3.15** |
| 66 and older | 1.04 | 0.67, 1.62 | **2.04** | **1.08, 3.87** | **1.73** | 0.96, 3.12 |
| **Sex** |  |  |  |  |  |  |
| Male | — | — | — | — | — | — |
| Female | 0.97 | 0.79, 1.20 | 1.07 | 0.84, 1.35 | 1.07 | 0.85, 1.33 |
| **Language region** |  |  |  |  |  |  |
| German / Romansch | — | — | — | — | — | — |
| French | 0.83 | 0.65, 1.07 | 0.95 | 0.71, 1.27 | 1.00 | 0.76, 1.31 |
| Italian | 0.69 | 0.41, 1.17 | 0.86 | 0.49, 1.51 | 0.80 | 0.46, 1.37 |
| **Survey start year** |  |  |  |  |  |  |
| 2016 | — | — | — | — | — | — |
| 2017-2019 | 1.16 | 0.93, 1.43 | 1.19 | 0.94, 1.51 | 1.23 | 0.98, 1.53 |
| 2020 onwards | **1.63** | **1.22, 2.18** | **1.63** | **1.19, 2.25** | **1.56** | **1.16, 2.11** |
| **Has children** |  |  |  |  |  |  |
| No | — | — | — | — | — | — |
| Yes | 0.94 | 0.78, 1.14 | 0.85 | 0.67, 1.08 | 0.79 | 0.63, 0.99 |
| **Highest degree: (applied) university** |  |  |  |  |  |  |
| No | — | — | — | — | — | — |
| Yes | **1.26** | **1.03, 1.55** | 1.20 | 0.96, 1.51 | 1.19 | 0.96, 1.48 |
| **Civil status** |  |  |  |  |  |  |
| Not in a partnership | — | — | — | — | — | — |
| Partnership / married | 1.12 | 0.93, 1.35 | 0.91 | 0.69, 1.19 | 1.19 | 0.96, 1.48 |
| **Living situation** |  |  |  |  |  |  |
| Living alone, Single-parenting | — | — | — | — | — | — |
| Living with spouse, family, friends or community | 1.12 | 0.90, 1.40 | 1.24 | 0.92, 1.67 | 1.16 | 0.87, 1.53 |
| **Swiss citizenship** |  |  |  |  |  |  |
| No | — | — | — | — | — | — |
| Yes | 1.18 | 0.89, 1.58 | 1.24 | 0.88, 1.73 | 1.21 | 0.89, 1.65 |
| **Years since MS diagnosis** | 0.99 | 0.98, 1.00 | 0.99 | 0.97, 1.00 | 0.92 | 0.81, 1.05 |
| **MS Type** |  |  |  |  |  |  |
| RRMS | — | — | — | — | — | — |
| CIS | 1.10 | 0.61, 2.01 | 1.24 | 0.59, 2.69 | 1.17 | 0.64, 2.17 |
| PPMS | 0.88 | 0.63, 1.22 | 0.88 | 0.59, 1.32 | 0.93 | 0.64, 1.37 |
| SPMS / Transition | 1.01 | 0.78, 1.32 | 1.24 | 0.86, 1.78 | 1.21 | 0.86, 1.71 |
| **MS in relatives** |  |  |  |  |  |  |
| No | — | — | — | — | — | — |
| Yes | 0.96 | 0.75, 1.21 | 0.98 | 0.76, 1.26 | 0.96 | 0.75, 1.22 |
| **Symptoms: fatigue** |  |  |  |  |  |  |
| No | — | — | — | — | — | — |
| Yes | **0.79** | **0.65, 0.96** | **0.75** | **0.57, 0.99** | 0.83 | 0.64, 1.07 |
| **Symptoms: paresthesia** |  |  |  |  |  |  |
| No | — | — | — | — | — | — |
| Yes | 1.21 | 1.00, 1.46 | 1.26 | 0.99, 1.61 | **1.28** | **1.02, 1.62** |
| **Symptoms: depression** |  |  |  |  |  |  |
| No | — | — | — | — | — | — |
| Yes | 0.82 | 0.62, 1.08 | 0.96 | 0.69, 1.34 | 0.92 | 0.67, 1.25 |
| **SRDSS score** |  |  |  |  |  |  |
| SRDSS 0-3.5 | — | — | — | — | — | — |
| SRDSS 4-6.5 | 0.92 | 0.72, 1.18 | 1.06 | 0.75, 1.49 | 1.04 | 0.77, 1.48 |
| SRDSS >=7 | 0.72 | 0.48, 1.06 | 1.04 | 0.59, 1.83 | 1.14 | 0.65, 1.91 |
| **Symptom burden** |  |  |  |  |  |  |
| No symptoms | — | — | — | — | — | — |
| 1-3 symptoms | 0.93 | 0.69, 1.25 | 1.02 | 0.70, 1.48 | 0.90 | 0.63, 1.28 |
| 4-6 symptoms | 0.95 | 0.70, 1.30 | 1.08 | 0.69, 1.69 | 0.98 | 0.64, 1.51 |
| More than 7 symptoms | 0.82 | 0.60, 1.11 | 1.05 | 0.61, 1.79 | 0.91 | 0.55, 1.05 |
| **Receives disability insurance** |  |  |  |  |  |  |
| No | — | — | — | — | — | — |
| Yes | **0.75** | **0.61, 0.92** | 0.81 | 0.60, 1.09 | 0.83 | 0.63, 1.10 |
| **Currently drives** |  |  |  |  |  |  |
| No | — | — | — | — | — | — |
| Yes | **1.43** | **1.14, 1.81** | **1.38** | **1.04, 1.82** | **1.32** | **1.02, 1.71** |
| **Uses public transport** |  |  |  |  |  |  |
| No | — | — | — | — | — | — |
| Yes | **1.43** | **1.14, 1.80** | 1.39 | 0.90, 2.17 | 1.40 | 0.94, 2.10 |
| **Currently working** |  |  |  |  |  |  |
| No | — | — | — | — | — | — |
| Yes | **1.26** | **1.03, 1.53** | 0.96 | 0.74, 1.25 | 1.02 | 0.80, 1.31 |
| **Someone helped with survey** |  |  |  |  |  |  |
| No | — | — | — | — | — | — |
| Yes | **0.56** | **0.36, 0.87** | 0.62 | 0.37, 1.03 | 0.68 | 0.42, 1.08 |
