# Supplementary material for: Real-world patterns in remote longitudinal study participation: A study of the Swiss Multiple Sclerosis Registry
Source: PLOS Digit Health. 2024 Nov 6;3(11):e0000645. doi: 10.1371/journal.pdig.0000645 (PMC11540223; doi:10.1371/journal.pdig.0000645)
Supplement: S8 Table — (DOCX) [file pdig.0000645.s012.docx]

## **S8 Table**: Sensitivity analysis with alternate outcome variable, univariate and multivariable logistic regression, yearly retention until the end of the study period

| **Variable** | **Univariate** | | **Multivariable - Global** | | **Multivariable - Imputed** | |
| --- | --- | --- | --- | --- | --- | --- |
|  | **OR***^1^* | **95% CI***^1^* | **OR***^1^* | **95% CI***^1^* | **OR***^1^* | **95% CI***^1^* |
| **Age** |  |  |  |  |  |  |
| 18-35 | — | — | — | — | — | — |
| 36-45 | **1.68** | **1.20, 2.37** | **1.89** | **1.31, 2.74** | **1.76** | **1.23, 2.52** |
| 46-55 | **1.77** | **1.27, 2.47** | **1.94** | **1.33, 2.86** | **1.89** | **1.31, 2.75** |
| 56-65 | **2.06** | **1.41, 3.02** | **2.49** | **1.55, 4.02** | **2.34** | **1.48, 3.71** |
| 66 and older | 1.36 | 0.74, 2.40 | 1.68 | 0.79, 3.51 | 1.66 | 0.82, 3.30 |
| **Sex** |  |  |  |  |  |  |
| Male | — | — | — | — | — | — |
| Female | 1.20 | 0.93, 1.56 | **1.41** | **1.06, 1.87** | **1.32** | **1.02, 1.73** |
| **Language region** |  |  |  |  |  |  |
| German / Romansch | — | — | — | — | — | — |
| French | 0.74 | 0.54, 1.02 | 0.87 | 0.61, 1.22 | 0.86 | 0.61, 1.19 |
| Italian | 0.64 | 0.30, 1.23 | 0.72 | 0.33, 1.41 | 0.68 | 0.32, 1.33 |
| **Survey start year** |  |  |  |  |  |  |
| 2016 | — | — | — | — | — | — |
| 2017-2019 | **0.68** | **0.53, 0.87** | **0.71** | **0.55, 0.93** | **0.70** | **0.55, 0.91** |
| 2020 onwards | **0.68** | **0.48, 0.95** | **0.68** | **0.47, 0.97** | **0.70** | **0.49, 0.98** |
| **Has children** |  |  |  |  |  |  |
| No | — | — | — | — | — | — |
| Yes | 1.17 | 0.93, 1.47 | 0.99 | 0.75, 1.31 | 0.98 | 0.75, 1.28 |
| **Highest degree: (applied) university** |  |  |  |  |  |  |
| No | — | — | — | — | — | — |
| Yes | 1.02 | 0.79, 1.29 | 1.02 | 0.78, 1.32 | 1.03 | 0.80, 1.32 |
| **Civil status** |  |  |  |  |  |  |
| Not in a partnership | — | — | — | — | — | — |
| Partnership / married | 1.28 | 1.02, 1.61 | 1.04 | 0.76, 1.43 | 1.10 | 0.81, 1.50 |
| **Living situation** |  |  |  |  |  |  |
| Living alone / Single-parenting | — | — | — | — | — | — |
| Living with spouse / family / friends / community | 1.23 | 0.93, 1.63 | 1.25 | 0.88, 1.78 | 1.19 | 0.85, 1.68 |
| **Swiss citizenship** |  |  |  |  |  |  |
| No | — | — | — | — | — | — |
| Yes | **1.64** | **1.10, 2.51** | 1.43 | 0.94, 2.22 | 1.39 | 0.95, 2.09 |
| **Years since MS diagnosis** |  |  |  |  |  |  |
| **MS type** |  |  |  |  |  |  |
| RRMS | — | — | — | — | — | — |
| CIS | 1.15 | 0.48, 2.52 | 1.55 | 0.62, 3.53 | 1.13 | 0.51, 2.30 |
| PPMS | 0.97 | 0.64, 1.44 | 0.98 | 0.61, 1.54 | 1.03 | 0.66, 1.58 |
| SPMS / Transition | 1.29 | 0.93, 1.75 | **1.57** | **1.05, 2.35** | **1.60** | **1.09, 2.34** |
| **MS in relatives** |  |  |  |  |  |  |
| No | — | — | — | — | — | — |
| Yes | 1.11 | 0.83, 1.46 | 1.14 | 0.85, 1.52 | 1.12 | 0.84, 1.47 |
| **Symptoms: fatigue** |  |  |  |  |  |  |
| No | — | — | — | — | — | — |
| Yes | 0.90 | 0.71, 1.13 | 0.81 | 0.59, 1.10 | 0.85 | 0.63, 1.14 |
| **Symptoms: paresthesia** |  |  |  |  |  |  |
| No | — | — | — | — | — | — |
| Yes | 1.21 | 0.96, 1.52 | 1.15 | 0.87, 1.53 | 1.19 | 0.91, 1.55 |
| **Symptoms: depression** |  |  |  |  |  |  |
| No | — | — | — | — | — | — |
| Yes | 0.72 | 0.49, 1.03 | 0.83 | 0.55, 1.23 | 0.79 | 0.53, 1.16 |
| **Symptom burden** |  |  |  |  |  |  |
| No symptoms | — | — | — | — | — | — |
| 1-3 symptoms | 1.24 | 0.86, 1.82 | 1.26 | 0.82, 1.95 | 1.12 | 0.74, 1.69 |
| 4-6 symptoms | 1.39 | 0.95, 2.05 | 1.47 | 0.88, 2.47 | 1.24 | 0.76, 2.03 |
| More than 7 symptoms | 1.08 | 0.74, 1.60 | 1.20 | 0.65, 2.22 | 1.02 | 0.56, 1.83 |
| **SRDSS score** |  |  |  |  |  |  |
| SRDSS 0-3.5 | — | — | — | — | — | — |
| SRDSS 4-6.5 | 1.17 | 0.88, 1.56 | 1.10 | 0.75, 1.61 | 1.04 | 0.72, 1.51 |
| SRDSS >=7 | **0.54** | **0.29, 0.92** | 0.51 | 0.24, 1.03 | 0.52 | 0.26, 1.00 |
| **Receives disability insurance** |  |  |  |  |  |  |
| No | — | — | — | — | — | — |
| Yes | 0.94 | 0.73, 1.21 | 0.91 | 0.65, 1.27 | 0.96 | 0.70, 1.33 |
| **Currently drives** |  |  |  |  |  |  |
| No | — | — | — | — | — | — |
| Yes | 1.22 | 0.91, 1.64 | 1.08 | 0.79, 1.50 | 1.07 | 0.79, 1.46 |
| **Uses public transport** |  |  |  |  |  |  |
| No | — | — | — | — | — | — |
| Yes | 1.46 | 0.96, 2.30 | 1.43 | 0.85, 2.47 | 1.29 | 0.80, 2.10 |
| **Currently working** |  |  |  |  |  |  |
| No | — | — | — | — | — | — |
| Yes | 1.05 | 0.82, 1.34 | 0.99 | 0.73, 1.34 | 0.99 | 0.74, 1.32 |
| **Someone helped with survey** |  |  |  |  |  |  |
| No | — | — | — | — | — | — |
| Yes | 0.68 | 0.36, 1.19 | 0.74 | 0.38, 1.36 | 0.75 | 0.40, 1.33 |
