# Supplementary material for: Real-world patterns in remote longitudinal study participation: A study of the Swiss Multiple Sclerosis Registry
Source: PLOS Digit Health. 2024 Nov 6;3(11):e0000645. doi: 10.1371/journal.pdig.0000645 (PMC11540223; doi:10.1371/journal.pdig.0000645)
Supplement: S9 Table — (DOCX) [file pdig.0000645.s013.docx]

## **S9 Table**: Sensitivity analysis with therapy timing variable, univariate and multivariable logistic regression, starting year-based retention

| **Variable** | **Univariate** | | **Multivariable - Global** | | **Multivariable - Imputed** | |
| --- | --- | --- | --- | --- | --- | --- |
|  | **OR***^1^* | **95% CI***^1^* | **OR***^1^* | **95% CI***^1^* | **OR***^1^* | **95% CI***^1^* |
| **Age** |  |  |  |  |  |  |
| 18-35 | — | — | — | — | — | — |
| 36-45 | **1.52** | **1.17, 1.98** | **2.10** | **1.54, 2.88** | **1.85** | **1.38, 2.48** |
| 46-55 | **1.50** | **1.16, 1.94** | **2.13** | **1.54, 2.96** | **1.95** | **1.43, 2.66** |
| 56-65 | **1.42** | **1.05, 1.94** | **2.13** | **1.40, 3.27** | **2.06** | **1.39, 3.08** |
| 66 and older | 1.04 | 0.67, 1.62 | **1.97** | **1.03, 3.81** | **1.67** | 0.92, 3.01 |
| **Sex** |  |  |  |  |  |  |
| Male | — | — | — | — | — | — |
| Female | 0.97 | 0.79, 1.20 | 1.06 | 0.84, 1.36 | 1.06 | 0.85, 1.33 |
| **Language region** |  |  |  |  |  |  |
| German / Romansch | — | — | — | — | — | — |
| French | 0.83 | 0.65, 1.07 | 0.96 | 0.72, 1.30 | 0.88 | 0.67, 1.16 |
| Italian | 0.69 | 0.41, 1.17 | 0.89 | 0.50, 1.57 | 0.84 | 0.48, 1.44 |
| **Survey start year** |  |  |  |  |  |  |
| 2016 | — | — | — | — | — | — |
| 2017-2019 | 1.16 | 0.93, 1.43 | 1.18 | 0.94, 1.50 | 1.25 | 1.00, 1.56 |
| 2020 onwards | **1.63** | **1.22, 2.18** | **1.68** | **1.21, 2.35** | **1.60** | **1.18, 2.17** |
| **Has children** |  |  |  |  |  |  |
| No | — | — | — | — | — | — |
| Yes | 0.94 | 0.78, 1.14 | 0.81 | 0.63, 1.04 | **0.78** | **0.62, 0.99** |
| **Highest degree: (applied) university** |  |  |  |  |  |  |
| No | — | — | — | — | — | — |
| Yes | **1.26** | **1.03, 1.55** | 1.15 | 0.91, 1.45 | 1.20 | 0.97, 1.49 |
| **Civil status** |  |  |  |  |  |  |
| Not in a partnership | — | — | — | — | — | — |
| Partnership / married | 1.12 | 0.93, 1.35 | 0.92 | 0.70, 1.21 | 1.07 | 0.82, 1.38 |
| **Living situation** |  |  |  |  |  |  |
| Living alone, Single-parenting | — | — | — | — | — | — |
| Living with spouse, family, friends or community | 1.12 | 0.90, 1.40 | 1.21 | 0.90, 1.64 | 1.16 | 0.87, 1.54 |
| **Swiss citizenship** |  |  |  |  |  |  |
| No | — | — | — | — | — | — |
| Yes | 1.18 | 0.89, 1.58 | 1.14 | 0.81, 1.60 | 1.21 | 0.89, 1.65 |
| **Years since MS diagnosis** | 0.99 | 0.98, 1.00 | 0.99 | 0.97, 1.00 | 0.92 | 0.81, 1.05 |
| **MS Type** |  |  |  |  |  |  |
| RRMS | — | — | — | — | — | — |
| CIS | 1.10 | 0.61, 2.01 | 1.36 | 0.62, 3.08 | 1.12 | 0.61, 2.06 |
| PPMS | 0.88 | 0.63, 1.22 | 0.80 | 0.53, 1.21 | 0.95 | 0.64, 1.41 |
| SPMS / Transition | 1.01 | 0.78, 1.32 | 1.24 | 0.85, 1.80 | 1.15 | 0.82, 1.63 |
| **MS in relatives** |  |  |  |  |  |  |
| No | — | — | — | — | — | — |
| Yes | 0.96 | 0.75, 1.21 | 1.01 | 0.78, 1.31 | 0.96 | 0.75, 1.22 |
| **Symptoms: fatigue** |  |  |  |  |  |  |
| No | — | — | — | — | — | — |
| Yes | **0.79** | **0.65, 0.96** | **0.75** | **0.56, 0.99** | 0.84 | 0.65, 1.10 |
| **Symptoms: paresthesia** |  |  |  |  |  |  |
| No | — | — | — | — | — | — |
| Yes | 1.21 | 1.00, 1.46 | 1.28 | 1.00, 1.63 | **1.27** | **1.01, 1.60** |
| **Symptoms: depression** |  |  |  |  |  |  |
| No | — | — | — | — | — | — |
| Yes | 0.82 | 0.62, 1.08 | 0.96 | 0.69, 1.34 | 0.91 | 0.66, 1.25 |
| **SRDSS score** |  |  |  |  |  |  |
| SRDSS 0-3.5 | — | — | — | — | — | — |
| SRDSS 4-6.5 | 0.92 | 0.72, 1.18 | 1.02 | 0.73, 1.45 | 1.04 | 0.75, 1.45 |
| SRDSS >=7 | 0.72 | 0.48, 1.06 | 1.03 | 0.58, 1.83 | 1.13 | 0.66, 1.94 |
| **Symptom burden** |  |  |  |  |  |  |
| No symptoms | — | — | — | — | — | — |
| 1-3 symptoms | 0.93 | 0.69, 1.25 | 1.06 | 0.72, 1.54 | 0.90 | 0.63, 1.27 |
| 4-6 symptoms | 0.95 | 0.70, 1.30 | 1.12 | 0.71, 1.76 | 0.98 | 0.64, 1.50 |
| More than 7 symptoms | 0.82 | 0.60, 1.11 | 1.10 | 0.64, 1.90 | 0.90 | 0.54, 1.49 |
| **Receives disability insurance** |  |  |  |  |  |  |
| No | — | — | — | — | — | — |
| Yes | **0.75** | **0.61, 0.92** | 0.78 | 0.58, 1.05 | 0.82 | 0.62, 1.08 |
| **Currently drives** |  |  |  |  |  |  |
| No | — | — | — | — | — | — |
| Yes | **1.43** | **1.14, 1.81** | **1.35** | **1.03, 1.79** | **1.34** | **1.04, 1.74** |
| **Uses public transport** |  |  |  |  |  |  |
| No | — | — | — | — | — | — |
| Yes | **1.49** | **1.10, 2.04** | 1.39 | 0.89, 2.17 | 1.41 | 0.94, 2.13 |
| **Currently working** |  |  |  |  |  |  |
| No | — | — | — | — | — | — |
| Yes | **1.26** | **1.03, 1.53** | 0.95 | 0.73, 1.24 | 1.02 | 0.80, 1.31 |
| **Someone helped with survey** |  |  |  |  |  |  |
| No | — | — | — | — | — | — |
| Yes | **0.56** | **0.36, 0.87** | 0.63 | 0.37, 1.05 | 0.73 | 0.45, 1.16 |
