# Supplementary material for: Real-world patterns in remote longitudinal study participation: A study of the Swiss Multiple Sclerosis Registry
Source: PLOS Digit Health. 2024 Nov 6;3(11):e0000645. doi: 10.1371/journal.pdig.0000645 (PMC11540223; doi:10.1371/journal.pdig.0000645)
Supplement: S10 Table — (DOCX) [file pdig.0000645.s014.docx]

## **S10 Table:** Descriptions of the complexity variables for SMSR surveys

| **Survey** | **Number of characters** | **Number of words** | **Number of questions** | **Fleisch-Kinkaid score** |
| --- | --- | --- | --- | --- |
| Follow-up survey 6 (FU6) | 28409 | 3589 | 75 | 8.02 |
| Follow-up survey 12 (FU12) | 30527 | 4025 | 75 | 8.06 |
| Follow-up survey 18 (FU18) | 38721 | 5208 | 79 | 6.88 |
| Follow-up survey 24 (FU24) | 30526 | 4049 | 64 | 7.28 |
| Follow-up survey 36 (FU36) | 45037 | 6749 | 132 | 5.17 |
| Finance thematic survey | 17698 | 2241 | 60 | 5.98 |
| COVID-19 (COV19) thematic survey | 2947 | 470 | 8 | 6.19 |
| COVID-20 (COV20) thematic survey | 11451 | 1535 | 38 | 6.41 |
| COVID-21 (COV21) thematic survey | 17752 | 2359 | 58 | 7.74 |
| Life Chart thematic survey | 42630 | 5415 | 155 | 7.18 |
