# Supplementary material for: Real-world patterns in remote longitudinal study participation: A study of the Swiss Multiple Sclerosis Registry
Source: PLOS Digit Health. 2024 Nov 6;3(11):e0000645. doi: 10.1371/journal.pdig.0000645 (PMC11540223; doi:10.1371/journal.pdig.0000645)
Supplement: Attachment — Submitted filename: 20240829_Rebuttal_Letter.pdf [file pdig.0000645.s015.pdf]

Editor's comments (summary of reviewers' comments)	Author response and changes made	Page number in revised paper
**Editor's comments**		
**Methods**<br><br>Make the Section more Compact:<br>Review each paragraph and sentence to eliminate redundancy.<br>Use concise language and remove any unnecessary details.<br>Consider using bullet points or subheadings for clarity.	Thank you for this comment. We proceeded to revise the **Methods** section of the manuscript to make the section more concise as suggested.	4, 5
**Methods**	Thank you for these important comments regarding the inclusion of clinical and socioeconomic variables that could potentially confound and impact survey completion rates.	
Addressing Factors Affecting the Model:<br>Underhoused Persons: Acknowledge the potential impact of underhoused individuals not completing follow-up surveys and how this might bias the results.	Thank you for this remark. While we acknowledge that underhousing can impact research participation, our study specifically focuses on data from Switzerland, which has one of the lowest rates of underhousing in the world. In Switzerland, estimates of the number of underhoused individuals ranges from 300 to 9000 individuals, which represent 0.003% to 0.1% of the Swiss population.[1] In the Swiss context, underhousing is predominantly associated with several mental illness and addiction[2], which are conditions not represented in the SMSR. Nevertheless, because the number of affected individuals is very low, we expect that our results on participation presented in this study are not biased.	
Hospitalized or Institutionalized Persons: Discuss the implications of participants being in hospitals or other facilities on survey completion rates.	Thank you for this remark. We agree that offering more context on these aspects would be helpful. In MS populations, we commonly observe two types of hospitalizations. The first common type is short-term hospitalization, where pwMS are admitted for acute exacerbations or relapses requiring immediate treatment, with a median hospitalization duration of 5 days.[3] In the context of the SMSR, it is difficult to capture these individuals, as the questionnaires can be completed at any time, with ongoing	17, 18

	participation not limited to a short period of time. Participants receive at least two reminders and have up to 6 months to fill them out. This means that even if participants received their first survey invitation while hospitalized, they have ample time outside of their hospitalization to complete the survey, along with receiving sufficient reminders.<br><br>The second common type is for participants in long-term care. In our baseline questionnaire, only 17 out of 1,757 participants (0.97%) were receiving long-term care. These numbers could illustrate the challenges of recruiting and enrolling participants who might have been in long-term care in the SMSR. As this population represents a very small subset of our study sample, we did not include them in the main analyses and do not believe that they may bias our results. We do, however, believe that future research could benefit from examining whether these aspects of care affect participation, especially if the timeframe provided to the participants does not offer them the flexibility to participate in the study outside of these settings. We proceeded to address this in the **Discussion** section as follows:<br><br>"*In the context of MS, considering additional contextual variables related to individuals' life and disease course could provide additional insights on long-term retention. For example, whether participants are receiving long-term care during the study could offer insights into their motivation to participate or their access to support for study participation. While we did not assess this due to the small size of this group in our study, it could be valuable to explore in future research.*"	
Timing of SMSR Completion: Clarify whether the SMSR is completed at the time of MS diagnosis or if it includes participants diagnosed over a prolonged period.	Thank you for this remark. Recruitment into the SMSR can occur at any timepoint after the confirmed diagnosis. As mentioned in the methods, participation in our Registry is not tied to a specific setting or treatment, which increases diversity when compared with clinic-based study settings.<br><br>We refer to this in the <u>Setting</u> subsection of the **Methods**:	4

	"*Participation in the SMSR is voluntary and requires completion of a signed consent form along with a confirmation of MS or clinically isolated syndrome (CIS) diagnosis. Enrollment can occur at any time point after diagnosis.*"<br><br>To account for time-related factors, such as the progression of MS since diagnosis, we already included the number of years since MS diagnosis in our analyses. Statistics on the number of years since MS diagnosis for our study sample are presented in **Table 1**.	
Discuss how MS treatments over time could affect the study results.	Thank you for this remark. As mentioned above, an advantage of the SMSR is that it recruits participants regardless of their treatment status. In turn, we find that participation in the SMSR is independent of MS treatment since pwMS can participate in the SMSR regardless of treatment status. Nevertheless, we agree with the reviewer that assessing MS treatments over time could provide additional context to our study results. A recent study by SMSR researchers found a shift towards early treatment with disease-modifying therapies, contrasting with the previous trend of delaying the start of these therapies.[4] In the former group, some participants may have cycled through all available treatments and are currently not undergoing treatment due to treatment resistance or an untreatable disease type. In contrast, the latter group may include participants who have had MS for a longer time but are still undergoing various treatment cycles.<br><br>The MS-related variables we included in our models may not be fully reflective of these trends. While participation in the SMSR is independent of MS treatment as reflected in our recruitment practices, we included a variable in the sensitivity analysis to evaluate whether participants 1) have ever had treatment, 2) are currently receiving treatment, or 3) are not receiving treatment, to reflect the trends mentioned earlier (**eTables 5** and **6**). We reflected this in the <u>Variables of interest</u> subsection of the **Methods** as follows:	5, 10, 13 (Main text); 15-18 (Appendix)

	"*Sensitivity analyses were performed to assess whether the timing of participants receiving disease-modifying therapies, or not receiving them at all, influenced the main analyses.*"<br><br>The sensitivity analysis showed no differences in the results of the models presented in the study. We mention these results in the **Results** section of the paper.<br><br><br>*****<br>**References**<br>1. Drilling M, Küng M, Dittmann J. Homelessness in Switzerland: Federalist Pathways Between Ignoring, Passing on Responsibility for, and Proactive Prevention of Homelessness. Routledge Handb Glob Perspect Homelessness Law Policy Routledge; 2024.<br><br>2. Drilling M, Küng M, Mühlethaler E, Dittmann J. Homelessness in Switzerland Understandings, policies and strategies of the cantons and communes. 2022. Available from: https://irf.fhnw.ch/server/api/core/bitstreams/9fb5701f-bf16-4edb-aca0-ac77bc690687/content [accessed Aug 19, 2024]<br><br>3. Maia C, Costa A, Abreu P, Sa MJ. All-cause hospitalizations in multiple sclerosis patients. Rev Neurol 2019 Mar 16;68(6):229–235. PMID:30855706<br><br>4. Barin L, Kaufmann M, Salmen A, Kamm CP, Gobbi C, Kuhle J, Pot C, Chan A, Czaplinski A, Ajdacic-Gross V, Rodgers S, Kesselring J, Puhan MA, von Wyl V. Patterns of care for Multiple Sclerosis in a setting of universal care access: A cross-sectional study. Mult Scler Relat Disord 2019 Feb 1;28:17–25. doi: 10.1016/j.msard.2018.11.033	
**Methods**	Thank you for this comment. Since the number of participants who left the SMSR due to death or de-registration represents a small subset of the study's sample (41 out of 1,757 participants,	3 (Appendix)

Managing Death or De-registration Events:<br>Explain how the departure of 41 patients due to death or de-registration was handled in the analysis.<br>Clarify whether these patients were classified as low retention or lower response than the median and how their data were treated.	approximately 2.3%), we allocated them to their respective retention outcomes just as we did for all other participants. We proceeded to include a table (**eTable 1**) in the appendix with the participation outcomes of this sample subset to provide context on whether their classification in regard to the main study sample.	
**Methods**<br><br>Justification for K-means Clustering Method:<br>Provide a rationale for choosing the K-means clustering method.<br>Compare it with other clustering methods such as Robust and Sparse K-means or weighted K-means, or justify why these methods were not used.	Thank you for this comment. We would like to highlight that the primary analyses of this study are the two logistic regressions, while the clustering analysis was more exploratory, serving as a supportive analysis to the main logistic regression analyses. We proceeded to highlight the exploratory nature of the clustering analysis to highlight this aspect in the Statistical analyses subsection of the **Methods** as follows:<br><br>"*We conducted an exploratory k-means clustering analysis to identify possible participant subgroups at baseline with varying participation.*"<br><br>We chose the K-means clustering method for several reasons. First, the data we used had already been cleaned for the logistic regressions, meaning it had no significant noise or outliers, which made the use of robust K-means unnecessary. We also had no underlying hypothesis suggesting that some data points were more important than others, which also did not make a weighted K-means a suitable approach for our analyses.<br><br>Overall, we found that standard K-means is well-suited for our data, making the algorithm efficient and straightforward to implement. Furthermore, standard K-means clustering is widely recognized and validated in similar research contexts that aim to identify subgroups in studies based on their participation, providing a reliable foundation for using K-means when no complex data structures or assumptions need to be accounted for. These factors together made K-means the optimal choice for our exploratory analysis.<br><br>Nevertheless, we agree with the reviewer that Sparse K-means could further strengthen the K-means analysis we present in our study by reducing the numbers of features used in the analysis,	5

	which could yield more meaningful clusters. We proceeded to conduct a sensitivity analysis of the original K-means clustering analysis with a sparse K-means approach. To address the feedback we received regarding the length of our methods section, and considering that the clustering analysis serves as a supportive exploratory analysis to the original logistic regressions, we chose not to include the results of the sensitivity analysis in the main manuscript. Rather, we proceeded to present them here in response to the reviewer's comment. We hope the reviewer agrees with our decision.<br><br>Overall, the results indicate three clusters that vary slightly in size compared to the initial analysis, however they reveal the same participant characteristics as in our initial analysis. The retention outcomes closely resemble those of the initial analysis, showing no significant differences in retention among the three clusters. The results are presented in **Supplemental Table 1** and **2** at the end of this rebuttal letter.	
**Methods**<br><br>Details on Unsupervised Clustering Methods:<br>Include more information about the unsupervised clustering methods and results.<br>Add the elbow method graphical representation in the supplemental information.	Thank you for this comment. We proceeded to rewrite the part of the clustering analysis in the Statistical analyses subsection of the **Methods** section for clarity. Nevertheless, in response to the other reviewer's suggestion to make the methods section more concise, and considering that the clustering analysis is not the primary focus of this study but was conducted as an exploratory analysis with a clean, low-dimensional dataset, we chose not to add further details. We hope that the reviewer understands and agrees with this choice.<br><br>We have added the results of the elbow method for our analysis in the appendix (**eFigure 2**).	4 (Main text),<br>3 (Appendix)
**Methods**<br><br>Validation of Clustering Analysis:<br>Explain any validation methods used for the unsupervised clustering analysis.<br>Discuss how the large sample size and multi-year span of the data were leveraged for validation.	Thank you for this comment. In our exploratory K-means clustering analysis, we did not use any validation methods. Validation methods are typically used in confirmatory analyses to rigorously assess the accuracy of clusters against known benchmarks, which was not the focus of our exploratory analysis. In our exploratory analysis, the focus was to support the main analyses by identifying potential subgroups of participants at	18

	baseline based on similarities in the data. At this stage, validation was not necessary as we were not aiming to rigorously test or confirm specific outcomes.<br><br>While formal validation methods were not used in the exploratory analysis, we find that the large sample size helps in informally validating the results. The greater number of data points provides a more comprehensive view of the data, allowing for more confident identification of patterns and clusters. Furthermore, in our analysis, the multi-year timespan did not impact the K-means clustering results because the clustering was performed solely on baseline variables. Since K-means is an unsupervised method, our analysis only considered the baseline data and is not affected by the longitudinal aspect of the study. After completing the clustering and extracting retention outcomes for participants in the identified clusters, the multi-year time span might have influenced the retention outcomes and differences among participants in the different clusters. However, no such differences were observed in our analysis.<br><br>Regarding the data itself used to conduct both the main analysis and the exploratory clustering analyses, we believe that future research should focus more on developing methods that include and assess more data that could affect participation rather than concentrating on the algorithm, such as K-means clustering. We proceeded to highlight this aspect in the **Discussion**:<br><br>"*As such, survey response patterns may be influenced by various unexplored factors. Therefore, instead of focusing on the analysis methods used to assess participation, attention should be directed toward developing objective metrics to predict participation based on new participant profiles and behaviors.*"	
**Results**<br><br>Improving Figure 3:<br>Make the figure more readable by increasing the size of the text.<br>Consider reformatting or redesigning the figure for better clarity.	Thank you for this comment. We have updated Figure 3 in the main manuscript to a version that is easier to read.	12

**References**<br><br>Consistent Formatting:<br>Ensure all references are formatted according to the journal's guidelines.<br>Use a consistent style for all references, including punctuation, capitalization, and order of information.	Thank you for this remark. We adapted the formatting of the references to meet the journal’s guidelines.	20-24
**Supporting Information**<br><br>Table of Contents:<br>Create a table of contents for the supporting information section, following the journal’s submission guidelines.	Thank you for this remark. We proceeded to include a table of contents for the supporting information document.	1 (Appendix)

Supplemental Material

Supplementary Table 1: Clustering task with Sparse K-means, baseline participants

Variable	Cluster 1 (n=595)	Cluster 2 (n=615)	Cluster 3 (n=392)
**Age**			
18-35	102 (17.1%)	266 (43.3%)	29 (7.4%)
36-45	210 (35.3%)	154 (25.0%)	57 (14.5%)
46-55	205 (34.5%)	141 (22.9%)	119 (30.4%)
56-65	63 (10.6%)	45 (7.3%)	130 (33.2%)
66 and older	15 (2.5%)	9 (1.5%)	57 (14.5%)
**Sex**			
Male	169 (28.4%)	129 (21.0%)	156 (39.8%)
Female	426 (71.6%)	486 (79.0%)	236 (60.2%)
**Language region**			
German / Romansch	470 (79.0%)	493 (80.2%)	307 (78.3%)
French	104 (17.5%)	100 (16.3%)	73 (18.6%)
Italian	21 (3.5%)	22 (3.6%)	12 (3.1%)
**Survey start year**			
2016	194 (32.6%)	161 (26.2%)	145 (37.0%)
2017-2019	299 (50.3%)	328 (53.3%)	202 (51.5%)
2020 onwards	102 (17.1%)	126 (20.5%)	45 (11.5%)
**Has children**			
No	144 (24.2%)	444 (72.2%)	148 (37.8%)
Yes	451 (75.8%)	171 (27.8%)	244 (62.2%)
**Highest degree: (applied) university**			
No	388 (65.2%)	404 (65.7%)	303 (77.3%)
Yes	207 (75.8%)	211 (34.3%)	89 (62.2%)
**Civil status**			
Not in a partnership	0 (0.00%)	615 (100%)	160 (40.8%)
Partnership / married	595 (100%)	0 (0.0%)	232 (59.2%)
**Living situation**			
Living alone / Single-parenting	3 (0.5%)	272 (44.2%)	83 (21.2%)
Living with spouse / family / friends / community	592 (99.5%)	343 (55.8%)	309 (78.8%)
**Swiss citizenship**			
No	66 (11.09%)	70 (11.4%)	37 (9.4%)
Yes	529 (88.91%)	545 (88.6%)	355 (90.6%)
**Years since MS diagnosis**	5.0 (11.0)	3.0 (9.0)	14.0 (14.0)
**MS type**			
RRMS	514 (86.4%)	541 (88.0%)	139 (35.5%)
CIS	7 (1.2%)	19 (3.1%)	4 (1.0%)
PPMS	37 (6.2%)	22 (3.6%)	90 (22.3%)
SPMS / Transition	37 (6.2%)	33 (5.4%)	159 (40.6%)
**MS in relatives**			
No	473 (79.5%)	492 (80.0%)	316 (80.6%)
Yes	122 (20.5%)	123 (20.0%)	76 (19.4%)
**Symptoms: fatigue**			
No	258 (43.4%)	280 (45.5%)	99 (25.3%)
Yes	337 (56.6%)	335 (54.5%)	293 (74.7%)
**Symptoms: paresthesia**			
No	311 (52.3%)	323 (52.5%)	161 (41.1%)
Yes	284 (47.7%)	292 (47.5%)	231 (58.9%)
**Symptoms: depression**			
No	542 (91.1%)	525 (85.4%)	332 (84.7%)
Yes	53 (75.8%)	90 (80.3%)	60 (35.2%)
**Symptom burden**			
No symptoms	101 (17.0%)	124 (20.2%)	3 (0.77%)
1-3 symptoms	223 (37.5%)	244 (39.7%)	33 (8.4%)
4-6 symptoms	147 (24.7%)	141 (22.9%)	122 (31.1%)
More than 7 symptoms	124 (20.8%)	106 (17.2%)	234 (59.7%)
**SRDSS score**			
SRDSS 0-3.5	593 (99.7%)	614 (99.8%)	0 (0.0%)

SRDSS 4-6.5	0 (0.0%)	0 (0.0%)	295 (75.3%)
SRDSS >=7	2 (0.34%)	1 (0.16%)	97 (24.7%)
**Receives disability insurance**			
No	489 (82.2%)	508 (82.6%)	140 (35.7%)
Yes	106 (17.8%)	107 (17.4%)	252 (64.3%)
**Currently drives**			
No	77 (12.9%)	107 (17.4%)	141 (36.0%)
Yes	518 (87.1%)	508 (82.6%)	251 (64.0%)
**Uses public transport**			
No	6 (1.0%)	4 (0.7%)	134 (34.2%)
Yes	589 (99.0%)	611 (99.3%)	258 (65.8%)
**Currently working**			
No	144 (24.2%)	121 (19.7%)	254 (64.8%)
Yes	451 (75.8%)	494 (80.3%)	138 (35.2%)
**Someone helped with survey**			
No	566 (95.1%)	598 (97.2%)	362 (92.4%)
Yes	29 (4.9%)	17 (2.8%)	30 (7.6%)

Supplementary Table 2: Retention outcomes based on identified clusters

Cluster	Retention Table	Percentage
Cluster 1 (n = 595)	Low retention	60.7
	High retention	36.3
	Lower response than median	43.5
	Higher response than median	55.4
Cluster 2 (n = 615)	Low retention	63.7
	High retention	39.3
	Lower response than median	44.6
	Higher response than median	56.5
Cluster 3 (n = 392)	Low retention	58.7
	High retention	41.3
	Lower response than median	48.2
	Higher response than median	51.8
